# The problem with good news: how should public health actors respond when alcohol consumption declines?

**DOI:** 10.1111/add.15018

**Published:** 2020-03-11

**Authors:** Victoria Whitaker, Melissa Oldham, Colin Angus, Hannah Fairbrother, John Holmes

**Affiliations:** ^1^ Health Sciences School University of Sheffield Sheffield UK; ^2^ School of Health and Related Research University of Sheffield Sheffield UK

**Keywords:** Advocacy, alcohol, policy, public debate, trends, youth drinking

There is a role for public health policy actors when alcohol consumption declines as trends may differ between population sub‐groups and harm may rise irrespective of consumption declines. In such circumstances, critical analysis of existing policies and emphasis on the need for policies informed by points of principle are needed.

Government alcohol policies can be categorised as remedial or structural in their fundamental orientation. Remedial policies are those that respond to marked worsening of public health trends (e.g. a sharp rise in liver disease deaths or rates of binge drinking). Remedial measures may include increasing alcohol duties and consequently prices, or enhanced resources for screening and brief intervention programmes targeted at high‐risk drinkers. In contrast, structural policies are those that address ongoing points of principle and are not reactions to fluctuating extant problems. Examples of structural policies may include preventing advertising of alcohol to children or prohibiting the sale of any alcohol at extremely low prices. We argue below that this distinction is particularly salient for public health policy actors in times when alcohol trends are improving.

Following steady increases across previous decades, per capita adult alcohol consumption declined in the UK by 18% between 2004 and 2016 1. Several other high‐income countries saw similar trends 2, signalling a potential reduction in the burden of harm from one of the key determinants of global ill‐health. However, for much of the same time‐period, public health actors in the countries affected argued that their governments' alcohol policies were orientated toward commercial interests, were not in line with the best available evidence and required strengthening to reduce alcohol‐related harm 3. This apparent mismatch between improving consumption trends and criticism of government action raises questions about whether public health actors' calls for stronger intervention were misplaced and, more generally, how these actors should respond when the prevalence of addictive behaviours declines.

Detailed analysis of alcohol‐related trends may justify continuing calls for further interventions when consumption is in decline. For example, the recent alcohol consumption decline in the UK is relative to an historic high in 2004 when the UK consumed more alcohol per capita than at any other point in almost 100 years 1, 4. Despite this decline, the UK remains one of the 20 heaviest drinking countries in the world 5. Moreover, the decline in alcohol consumption in the UK did not coincide with a decline in alcohol‐attributable harm. Instead, alcohol‐attributable hospitalisations and deaths increased 6, 7. This is striking, given that many argue that population‐level consumption and harm trends typically move in the same direction over time 8.

Notably marked disparities in consumption trends can emerge between age groups, partly explaining contrary trends in consumption and harm. While there has been a decline in youth drinking in many high‐income countries, there have also been concurrent increases in consumption amongst middle and older age groups 9. Despite the latter age groups typically accounting for the majority of alcohol‐related harm to health, their drinking tends not to be pathologised in the same way as youth drinking. Moreover, they are the focus of far fewer alcohol interventions than younger age groups 10 and, as such, there remains an impetus for public health interventions that are sensitive to the particularities of population sub‐groups even while consumption declines at the population level.

We should also be cautious of assuming that reductions in alcohol consumption will persist in the long‐term 11, and sceptical of claims that government alcohol policy has played a substantive role in those reductions. For example, there is little evidence to suggest a direct role for preventative policies in the international decline in youth drinking 12, 13, despite the claims of some industry organisations 14. Instead, alcohol consumption trends may be subject to influence from numerous interlinked social shifts.

Alcohol‐centric and other policies may be implicated in societal shifts without being the principal determinants of them 15. For example, the decline in youth drinking in the UK was preceded by more stringent attempts to govern alcohol supply (e.g. the Challenge 21 and 25 schemes that sought to prevent underage sales). However, these attempts coincided with several other important policies that may also be implicated in current youth drinking trends. Such policies include those that placed restrictions on young people's use of public spaces (e.g. 16 and promoted the engagement of more young people in tertiary education (e.g. 17). In the UK and internationally, a proliferation of new home entertainment technologies, economic uncertainties, and shifting societal perceptions of risk have reshaped the contexts in which youth practices relating to alcohol develop and play out. As a result, young people's everyday lives are now increasingly lived within domestic settings where they are often under adult supervision and may be less likely to drink or, when they do, to drink to intoxication. These trends are beyond the control of public health policy‐makers and advocates, and future shifts may change the situation to encourage increased youth drinking. Therefore, there remains a justification for advocating and adopting effective alcohol policies that would hinder such a reversal.

If public health policy actors are to continue in their efforts when consumption trends are improving, then reflection is still required on which recommendations and messages remain appropriate in ever‐changing policy contexts. One potential approach is to begin to distinguish between remedial policies and structural policies—although we recognise that, in practice, there may be no clear dichotomy between such types.

As consumption declines, we suggest that public health advocates should not ignore remedial policies but might give greater attention to structural approaches. Remedial policies are not diminished in the their importance by this revised focus of attention, but should target instead the demographic groups that continue to exhibit the most risky behaviours or which experience sustained high levels of harm. Where public health actors advocate structural policies, they should clearly assert that these are underpinned by points of principle, not a response to extant and/or worsening problems. This should help public health advocates' arguments to continue to have force when trends of concern begin to improve.

## Declaration of interests

None.

## Acknowledgements

This work was supported by the Wellcome Trust (grant number: 208090/Z/17/Z)
